# *Salmonella* Transiently Reside in Luminal Neutrophils in the Inflamed Gut

**DOI:** 10.1371/journal.pone.0034812

**Published:** 2012-04-06

**Authors:** Yvonne Loetscher, Andreas Wieser, Jette Lengefeld, Patrick Kaiser, Sören Schubert, Mathias Heikenwalder, Wolf-Dietrich Hardt, Bärbel Stecher

**Affiliations:** 1 Institute of Microbiology, ETH Zürich, Zürich, Switzerland; 2 Max von Pettenkofer Insititute, Ludwig Maximilians University Munich, Munich, Germany; 3 Institute for Virology, Technical University Munich/Helmholtz Center Munich, Munich, Germany; Indian Institute of Science, India

## Abstract

**Background:**

Enteric pathogens need to grow efficiently in the gut lumen in order to cause disease and ensure transmission. The interior of the gut forms a complex environment comprising the mucosal surface area and the inner gut lumen with epithelial cell debris and food particles. Recruitment of neutrophils to the intestinal lumen is a hallmark of non-typhoidal *Salmonella enterica* infections in humans. Here, we analyzed the interaction of gut luminal neutrophils with *S. enterica* serovar Typhimurium (*S.* Tm) in a mouse colitis model.

**Results:**

Upon *S.* Tm^wt^ infection, neutrophils transmigrate across the mucosa into the intestinal lumen. We detected a majority of pathogens associated with luminal neutrophils 20 hours after infection. Neutrophils are viable and actively engulf *S.* Tm, as demonstrated by live microscopy. Using *S.* Tm mutant strains defective in tissue invasion we show that pathogens are mostly taken up in the gut lumen at the epithelial barrier by luminal neutrophils. In these luminal neutrophils, *S.* Tm induces expression of genes typically required for its intracellular lifestyle such as siderophore production *iroBCDE* and the *Salmonella* pathogenicity island 2 encoded type three secretion system (TTSS-2). This shows that *S.* Tm at least transiently survives and responds to engulfment by gut luminal neutrophils. Gentamicin protection experiments suggest that the life-span of luminal neutrophils is limited and that *S*. Tm is subsequently released into the gut lumen. This “fast cycling” through the intracellular compartment of gut luminal neutrophils would explain the high fraction of TTSS-2 and *iroBCDE* expressing intra- and extracellular bacteria in the lumen of the infected gut.

**Conclusion:**

In conclusion, live neutrophils recruited during acute *S.* Tm colitis engulf pathogens in the gut lumen and may thus actively engage in shaping the environment of pathogens and commensals in the inflamed gut.

## Introduction

Non-typhoidal *Salmonella enterica* strains (NTS) cause a major fraction of world-wide bacterial infections. After oral ingestion of contaminated food, pathogens replicate in the gut lumen, invade mucosal tissues and trigger acute enteritis within hours after infection. Key pathological characteristics of human NTS infections are mucosal edema and acute inflammation with polymorph nuclear leukocyte (PMN) infiltration, PMN transmigration into the gut lumen and the formation of crypt abscesses [1], [2], [3], [4]. These typical features are also recapitulated in bovine and murine NTS colitis models [5], [6], [7], [8], [9]. However, the role of neutrophils for antimicrobial defense in the gut lumen has not been addressed so far. It remained unclear whether gut luminal neutrophils represent “inactive” cellular waste from the inflammatory mucosal defense or whether they can actively engage in the host-pathogen interaction.

The acute inflammatory response in the gut plays an important role for the pathogens’ lifestyle as it changes microbiota-pathogen competition in favor of the pathogen [10], [11]. In the absence of inflammatory conditions, the microbiota blocks pathogen colonization ( = colonization resistance) [12], [13]. By triggering mucosal inflammation, NTS can alter gut microbial ecology and thereby boost pathogen growth in the gut lumen [14], [15]. Similar observations have been made for other enteric pathogens such as *Citrobacter rodentium* as well as for *Clostridium difficile* [16], [17]. We are just beginning to unravel the mechanisms explaining the growth benefit of NTS in the inflamed gut.

*Salmonella enterica* serovar Typhimurium (*S.* Tm) seems to be extremely well adapted to life in the inflamed gut. *S.* Tm can metabolize sugars released by the inflamed mucosa, which it can access via flagella-mediated chemotaxis [18], [19]. Furthermore, it can utilize tetrathionate as an electron acceptor for anaerobic respiration. Tetrathionate is generated via phagocyte oxidative burst from thiosulfate and is therefore only available upon inflammation. Thus, in the oxygen limited environment of the gut, NTS (which can respire tetrathionate) can generate energy more effectively than most commensals which cannot respire tetrathionate and have to rely on fermentation [20].

Besides its capacity to efficiently exploit energy sources available in the inflamed gut, when compared to the majority of commensal bacteria, *S.* Tm is also more resistant to a variety of host defenses activated in the inflamed gut. *S.* Tm harbors *iroBCDE* and *iroN*, a genetic locus for salmochelin synthesis and uptake [21]. Salmochelin is a glycosylated form of the common enterobacterial siderophore enterochelin. However, in contrast to enterochelin, salmochelin is resistant to binding and inactivation by the hosts antimicrobial peptide lipocalin-2, which is upregulated in the inflamed gut [22]. Moreover, in contrast to a variety of commensal bacteria, *S.* Tm is resistant against RegIIIβ, an antimicrobial lectin produced upon *Salmonella*-induced colitis in mice [23]. Overall, *S.* Tm seems to capitalize on diverse features of the inflamed gut in order to colonize the hosts intestine and to out-compete the resident microbiota. However, the inflamed gut is a highly structured environment which offers distinct niches to be exploited for bacterial growth. The niches include the mucosal surface, the mucus layer and the inner gut lumen containing shed epithelial cells and food particles. It has remained unclear whether and how these different niches may support pathogen growth.

In this study, we set out to characterize the interaction of *S.* Tm with neutrophils that infiltrate the gut lumen in response to infection. We show that most *S.* Tm are engulfed by neutrophils right in the gut lumen and reside for a limited time within these host cells. Our data suggest that neutrophils should be considered as an additional intracellular compartment for residence and/or replication of pathogens in the gut lumen.

## Results

### In the Inflamed Gut Lumen, *S.* Tm is Associated with CD18^+^ Neutrophils

**Figure 1:**
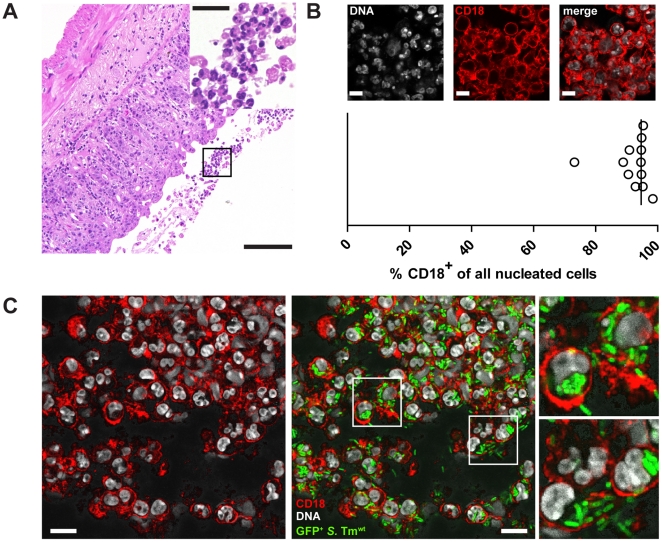
*S.* Tm is associated with CD18^+^ neutrophils in the gut lumen. **A.** H&E stained section of cecum tissue obtained from streptomycin-treated C57Bl/6 mouse infected with *S.* Tm^wt^ for 20 h. Polymorphonuclear cells are detected in the mucosa as well as in the gut lumen (inset). Scale bar: 100 µm; inset: 20 µm; L =  lumen. **B.C.** Immunofluorescence staining of gut lumina of *S.* Tm infected mice. Mice (n = 4) were treated with streptomycin and infected with *S.* Tm^wt^ pM979 for 20 h. Tissue was fixed and stained with an α-CD18 antibody. Confocal images were obtained and the fraction of CD18^+^ cells of all nucleated cells in the gut lumen was determined (3 independent images/mouse). Scale bar: 10 µm (**B**). GFP^+^ *S.* Tm co-localize with CD18^+^ cells in the gut lumen Scale bar: 10 µm. GFP (green), CD18 (red) and DNA/nuclei (Sytox-green, grey) (**C**).

Transmigration of neutrophils into the gut lumen is a cardinal feature of acute *S.* Tm triggered gut inflammation. However, the interaction of neutrophils with pathogens in the gut lumen has not been addressed so far. In the streptomycin colitis model, neutrophil infiltration and transmigration can be reliably observed at 20h post infection with *S.* Tm^wt^ (**Fig. 1A** **;** **Table 1**). CD18^+^ neutrophils constitute the majority of nucleated cells in the gut lumen 20h post infection (**Fig.1B**). At this time point, *S.* Tm colonizes the gut lumen at high density (>10^9^ cfu/g; [14]). To analyze *S.* Tm interaction with transmigrated neutrophils we performed immunfluorescence microscopy of cecal tissues of mice infected with *gfp*-expressing *S.* Tm^wt^ (*S.* Tm^wt^ p*rpsM*-*gfp*). Numerous GFP-positive bacteria were detected in association with CD18^+^ cells (**Fig.1C** **)**.

**Table 1:** *S.* Tm strains and plasmids.

*S.* Tm *strains*	*Genotype*	*Reference*
*S.* Tm^wt^(SB300)	*S.* Tm strain SL1344	[39]
*S.* Tm^avir^(M557)	*S.* Tm Δ *invG*; *ssaV*::*aphT*	[25]
*S.* Tm*^fliGHI^*(M913)	*S.* Tm *fliGHI*::Tn10	[18]
*S.* Tm*^cheY^*(M935)	*S.* Tm *cheY*::Tn10	[18]
*S.* Tm*^avir fliGHI^*(M933)	*S.* Tm *fliGHI*::Tn10; Δ *invG*; *ssaV*::*aphT*	[19]
M960	*S.* Tm Δ *invG*; *ssaV*::*aphT*. Histidin prototrophic variant by P22-transduction from S. Tm ST4/74 [40]	[19]
***Plasmids***		
p*rpsM-gfp*(pM979)	p*rpsM-gfp*	[18]
p*ssaG-gfp* (pM975)	p*ssaG-gfp*	[28]
p*iroBCDE-gfp* (pM1445)	p*iroBCDE-gfp*	This study
p*sodB-gfp*(pM1446)	p*sodB-gfp*	This study
ptYFP turbo YFP	Constitutive *yfp* expression	[26]

**Figure 2:**
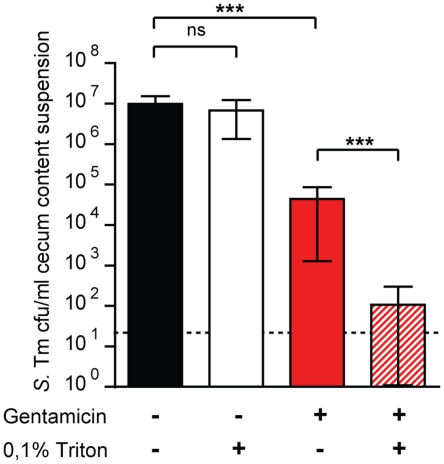
Luminal *S.* Tm are gentamicin-protected. Gentamicin-protection assay of gut luminal *S.* Tm. Streptomycin-treated mice (n = 9) were infected with *S.* Tm^wt^. 20h post infection, the mice were sacrificed and cecum contents collected in PBS and split into 4 fractions (untreated; treated with triton-X-100 (0.1%) for 30 minutes; treated with gentamicin (400 µg /ml) for 30 minutes; or treated with triton-X-100 (0.1%) and gentamicin (400 µg/ml) for 30 minutes). Samples were diluted and plated on McConkey agar plates (100 µg/ml streptomycin), allowing specific detection of *S.* Tm. Bars show mean and StD of *S.* Tm^wt^ CFU/ml; xxx  =  p < 0.0001.

To assess if live *S.* Tm^wt^ is contained within intact neutrophils, we performed a gentamicin protection assay on cecal content 20h post infection with *S.* Tm^wt^ (9 mice in total). In this type of experiment, bacteria contained in eukaryotic cells having an intact membrane are protected from gentamicin-mediated killing since the antibiotic does not enter intact cells. The luminal content was split into 4 fractions. The fractions were either treated with triton-X-100 (0.1%) to lyse all intact host cells or with gentamicin to kill all extracellular bacteria or with both (triton-X-100 and gentamicin) to kill all bacteria, or left untreated, as control. We used a high gentamicin concentration (400 µg/ml) to ensure that all extracellular *S.* Tm are killed efficiently in the heterogeneous luminal content. As anticipated, triton-X-100 alone did not lead to a significant reduction in total *S.* Tm^wt^ numbers (**Fig. 2**). This is in line with the resistance of NTS against many detergents. Interestingly, approximately 1% of the luminal bacteria were protected from gentamicin (0,62 ± 0,64%; p<0.0001), suggesting that viable *S.* Tm reside within host cells. In line with this, host cellular lysis by triton-X-100 further reduced the fraction of gentamicin-protected bacteria by more than 100-fold (0,0015% ± 0,002%, p<0.0001). In conclusion, a small fraction of *Salmonella* residing in the gut lumen is viable and protected from gentamicin and therefore supposedly intracellular (i.e. within CD18^+^ cells). In combination with the data presented in Fig. 1, our data suggested that CD18^+^ neutrophils represent a discrete intracellular compartment in the lumen of the inflamed gut which contains live pathogens.

### Life Microscopy Shows *S.* Tm^avir^ Uptake by Active Neutrophils in the Gut Lumen

**Figure 3:**
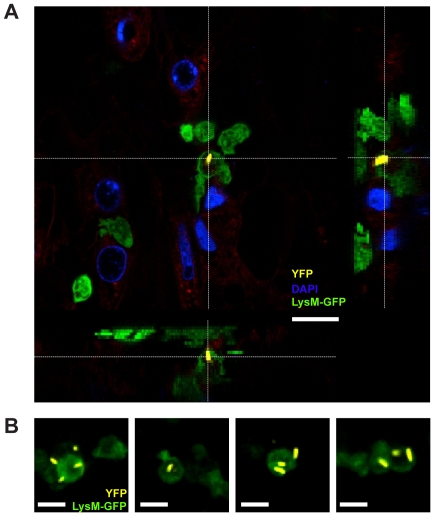
Luminal neutophils are viable and actively engulf *S.* Tm. Streptomycin-treated mice LysM^gfp^ +/− mice were infected with *S.* Tm^wt^ and sacrificed 24h post infection. The cecum was explanted and infected *ex vivo* with *yfp* expressing *S.* Tm^avir^, stained with DAPI (DNA; blue) and FM 4–64 (epithelial membranes; red), mounted on an organ holder and analyzed by confocal real time microscopy of *S.* Tm and GFP^+^ neutrophils. **A.** Representative confocal image stack showing *yfp* expressing *S.* Tm^avir^ within a GFP^+^ neutrophil. **B.** Representative confocal images showing live GFP^+^ neutrophils associated with *S.* Tm^avir^. Scale bars: 10µm.

To actively take up *S.* Tm within the cecum lumen after transmigration, neutrophils should be viable and phagocytic. To test this, we performed time lapse microscopy of neutrophils from explanted, *S.* Tm^wt^ infected ceca of LysM^gfp+/-^ mice [24]. In these mice, the *egfp* gene is knocked into the murine lysozyme M locus and leads to *egfp* expression particularly in mature, neutrophilic granulocytes [24]. Streptomycin-treated LysM^gfp+/−^ mice were infected with *S.* Tm^wt^ for 20 hours to induce neutrophil transmigration. The cecum was explanted, flushed carefully and infected *ex vivo* with *yfp*-expressing *S.* Tm^avir^ (*ΔinvGssaV::aphT* [25]). We used *S.* Tm^avir^, which lacks functional TTSS−1 and −2 type III secretion systems, to exclude that the bacteria could actively invade the neutrophils. In this way, we could focus our analysis on active bacterial uptake by the neutrophils. To visualize the rest of the mucosal tissue (besides the pathogen and the neutrophils), we included DAPI and the lipophilic styryl dye FM 4–64 into the assay buffer and mounted the organ as described [26]. Luminal neutrophils were imaged by confocal time-lapse microscopy as described [26]. We observed motile Egfp-positive neutrophils within gut lumen (**Movies S1, S2**). Furthermore, we frequently detected Yfp-positive *S.* Tm^avir^ contained within LysM^gfp+/−^ cells in the gut lumen (**Fig. 3AB**). This demonstrated that gut luminal neutrophils are viable at least for a certain time after transmigration and that they can actively engulf *S.* Tm in the gut lumen.

### *S.* Tm Uptake by Neutrophils Occurs in the Gut Lumen and is Fostered by TTSS-Independent, Flagella-Mediated Chemotaxis

**Figure 4:**
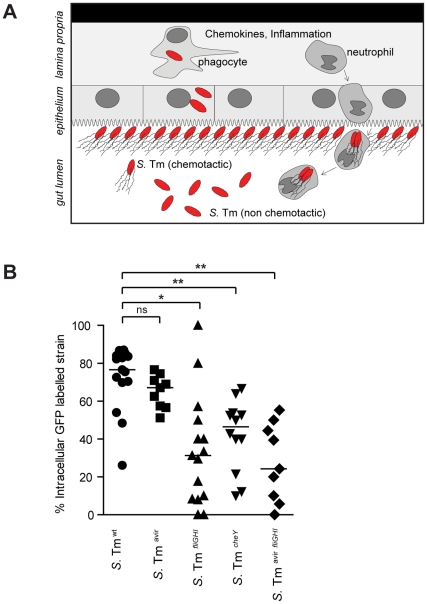
Transmigrating neutrophils engulf *S.* Tm in the gut lumen. **A.** Model for the interaction of transmigrating PMNs with luminal *S.* Tm. *S.* Tm enrich at the epithelial surface via flagella-mediated chemotaxis. PMNs exiting from the mucosa would engulf first those bacteria close to the epithelial border before migrating further into the center of the intestinal lumen. **B.** Streptomycin-treated mice (n = 3–5) were co-infected with *S.* Tm^wt^ and GFP-labeled *S.* Tm mutant strains carrying pM979 for constitituve *gfp*-expression (*S.* Tm^wt^ *; S.* Tm^avir^; *S.* Tm*^fliGHI^*; *S.* Tm*^cheY^*; *S.* Tm^avir *fliGHI*^). 20h post infection, the mice were sacrificed and cecum prepared for immunofluorescence staining of extracellular *S.* Tm with α-*Salmonella*-O-antigen group B serum as described in the materials and methods section. The fraction of intracellular GFP^+^ *S.* Tm was determined as (GFP^+^ LPS^−^/ GFP^+^ LPS^+^) × 100%.

As demonstrated above, pathogens can be directly taken up by transmigrated, actively phagocytic neutrophils within the gut lumen (**Fig. 2** **;** **Fig. 4A**). Alternatively (or additionally), bacteria could be taken up by neutrophils in the mucosal tissue and subsequently exported into the gut lumen. To distinguish between these different possibilities, we performed the following experiment. Streptomycin-treated mice were co-infected for 20h with a mixture of 50% *S.* Tm^wt^ and 50% *gfp*-expressing *S.* Tm^wt^ (p*rpsM*-*gfp*; positive control group) or a mixture of 50% *S.* Tm^wt^ and 50% *S.* Tm^avir^ (p*rpsM*-*gfp*; experimental group). *S.* Tm^avir^ (p*rpsM*-*gfp*) neither invades mucosal tissues, nor replicates in the lamina propria, and thus is restricted mostly to the gut lumen.

By immunfluorescence microscopy of fixed tissue sections we differentiated between extra- and intracellular *S.* Tm and determined the fraction of intracellular *gfp*-expressing *Salmonella spp* in the gut lumen. Extracellular *gfp*-expressing *S.* Tm were co-stained with an anti-*Salmonella* LPS antiserum (GFP^+^; LPS^+^) while intracellular *S.* Tm were not stained (GFP^+^; LPS^-^). We found a similar fraction (72,3 ± 17,2%) of *S.* Tm^wt^ and *S.* Tm^avir^ (65,1 ± 8,7%) located within luminal neutrophils (**Fig. 4B**). This strongly argues for the fact that the majority of pathogens is taken up by transmigrated neutrophils within the gut lumen. However, it should be noted that this microscopic assay cannot distinguish between life and dead neutrophils. This may explain why only a small fraction of the neutrophil-associated bacteria are efficiently protected from gentamicin (see Fig. 2).

We have shown before that *S.* Tm requires flagella-mediated chemotaxis to swim towards the epithelium [18]. In infected mice, flagellated, chemotactic bacteria are significantly enriched at the epithelium compared to the center of the gut lumen [19]. We took advantage of this fact and analyzed a *S.* Tm flagella mutant (*S.* Tm*^fliGHI^* p*rpsM*-*gfp*), a chemotaxis mutant (*S.* Tm*^cheY^* p*rpsM*-*gfp*) or an avirulent, chemotaxis mutant (*S.* Tm*^avir fliGHI^* p*rpsM*-*gfp*) with regard to the fraction of luminal host-cell associated bacteria. The experiment was done in analogy to the one described above. We hypothesized, that neutrophils exiting the mucosa would first take up bacteria localized directly at the epithelium, a site reached by the pathogen in a flagella- and chemotaxis-dependent fashion (**Fig. 4A**). Indeed, a significantly lower fraction of *S.* Tm*^fliGHI^, S.* Tm*^cheY^* and *S.* Tm*^avir fliGHI^* was detected within neutrophils when compared to flagellated, chemotactic strains (**Fig. 4B**). This finding supports our hypothesis, that neutrophils engulf luminal *S.* Tm after transmigration into the gut lumen. Additionally, flagella and chemotactic motility may support engulfment by PMNs.

Again, the assay cannot distinguish between life and dead luminal neutrophils. This may explain the apparent difference to the data shown in Fig. 2. In the gentamicin-protection assay we only found approx. 1% of the luminal bacteria to be gentamicin-protected (**Fig. 2**). This number is much smaller than the fraction of bacteria associated with neutrophils (**Fig. 4**) and may be due to the fact, that most luminal neutrophils are already dead and permeable during the gentamicin treatment (but not permeable to the α-LPS antibody). In this case, intracellular containment of *S.* Tm in luminal PMNs would only be a transient stage for *S.* Tm in the gut lumen. Alternatively, the majority of pathogens are killed upon neutrophil engulfment and neutrophils would thus be a “dead-end” for luminal *S.* Tm.

### Expression of the SPI-2 TTSS is Upregulated by Intra- and Extracellular *S.* Tm in the Inflamed Gut Lumen

**Figure 5:**
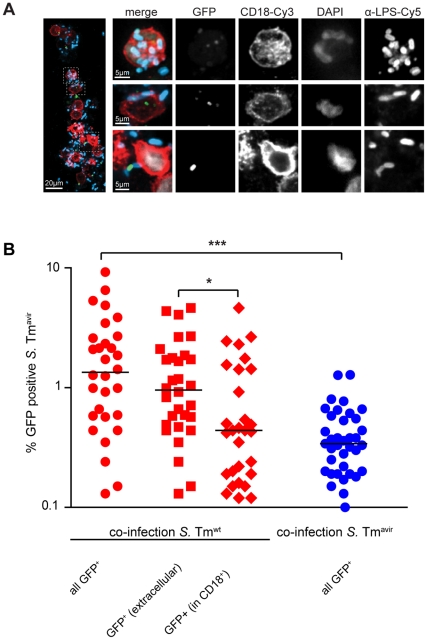
*Salmonella* induce expression of the SPI-2 TTSS in neutrophils in the inflamed intestine. 2 groups of streptomycin-treated mice (n = 3) were co-infected with *S.* Tm^avir^ containing the GFP-reporter plasmid p*ssaG*-*gfp* and either *S.* Tm^wt^ (red; inflammation induced) or *S.* Tm^avir^ (blue; no inflammation induced). 20h post infection, the mice were sacrificed and cecum prepared for immunofluorescence staining of *S.* Tm with α-*Salmonella*-O-antigen group B serum and anti-CD18 as described in the materials and methods section. The tissue was not treated with triton. **A**. Representative images of intra- and extracellular *S.* Tm^avir^ p*ssaG*-*gfp* in the cecal lumen. Scale bar: 20µm and 5 µm. α-*Salmonella* LPS antiserum (blue), CD18 (red) and GFP (green). **B.** Quantitative image analysis of % *gfp*-expressing of all LPS^+^ *S.* Tm in the inflamed and non-inflamed gut. In the inflamed gut, the % of extracellular (GFP^+^ LPS^+^) and intracellular (GFP^+^ LPS^−^ and CD18-associated) was determined as well. Bars show the median.

In order to distinguish whether *S.* Tm live or die within the luminal neutrophils, we set up an experiment to localize *S.* Tm that have adapted to an intracellular lifestyle in the gut lumen. The pathogenicity island 2 TTSS (TTSS-2) is one of the most widely used reporters for the intracellular stage of *S.* Tm [27], [28], [29]. We co-infected streptomycin-treated mice (n = 3) with an avirulent *S.* Tm strain carrying a reporter for TTSS-2 expression (*S.* Tm^avir^ p*ssaG*-*gfp*; no tissue invasion) with equal amounts of unlabeled *S.* Tm^avir^ (n = 3; no inflammation) or unlabeled *S.* Tm^wt^ (n = 3; inflammation). We compared the percentage of *S.* Tm^avir^ expressing the TTSS-2-reporter under non-inflamed and inflamed conditions by fluorescence microscopy (**Fig. 5AB**). We found a significant increase of TTSS-2-reporter expression in the inflamed gut (red symbols) versus the non-inflamed cecal lumen (blue symbols; median 1,34% versus 0,34%). This suggested, that a higher fraction of luminal *S.* Tm^avir^ adapts to an intracellular lifestyle in the inflamed gut.

Next, we analyzed *gfp*-expressing *S.* Tm^avir^ with respect to their localization in the inflamed gut. Approx. 2/3 of this population was located extracellularly (median 0.96%; co-stained with the α-LPS antiserum, not associated with CD18^+^ cells) and approx. 1/3 was located in association with CD18^+^ neutrophils (median 0,44%; not stained with the α-LPS antiserum, contained in CD18^+^ cells). These data lends further support to the notion that *S.* Tm is just transiently contained within CD18^+^ cells in the gut lumen. This stage lasts long enough to respond with *ttss-2* expression. In contrast, the relatively high fraction of TTSS−2^+^ extracellular *S.* Tm indicates that these bacteria are set free at a certain stage (i.e. upon death/lysis of the neutrophil).

Taken together, our data suggest that at least part of the gut luminal pathogen population is transiently contained within CD18^+^ cells and that the intracellular phase is long enough to adapt to an intracellular lifestyle.

### *S.* Tm Contained within Luminal Neutrophils Induce Expression of *iroBCDE*

Finally, we wanted to more closely analyze the environment of neutrophil-contained *S.* Tm. Raffatellu et *al.* have recently shown that *S.* Tm circumvents the host antimicrobial peptide lipocalin-2, which sequesters the siderophore enterochelin and is induced in the inflamed gut. Lipocalin-2 is expressed by epithelial cells [22] as well as neutrophils [30]. *S.* Tm harbors a gene cluster for production of the siderophore salmochelin and its cognate receptor IroN (*iroBCDE iroN*) which is not inhibited by lipocalin-2. However, it remained unclear whether enterochelin-mediated iron uptake is expressed by bacteria freely in the gut lumen or while the pathogen resides within gut luminal neutrophils.

**Figure 6:**
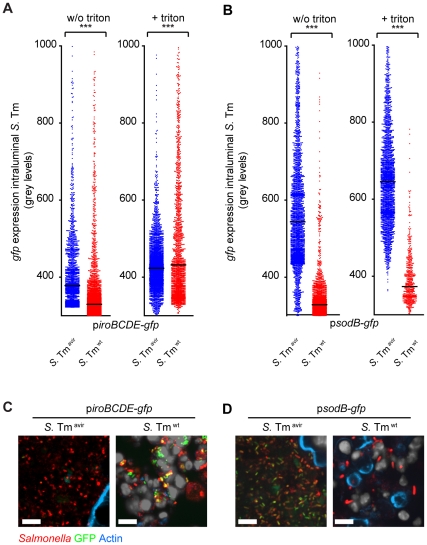
*Salmonella* contained within luminal neutrophils experience iron limitation. 4 groups of streptomycin-treated mice (n = 3) were infected with *S.* Tm^wt^ (red; inflammation induced) or *S.* Tm^avir^ (blue; no inflammation induced) containing the GFP-reporter plasmids p*iroBCDE*-*gfp* (**A, C**) or p*sodB*-*gfp* (**B, D**), respectively. 20 h post infection, the mice were sacrificed and cecum prepared for immunofluorescence staining of *S.* Tm with α-*Salmonella*-O-antigen group B serum as described in the materials and methods section. The tissue was either not permeabilized (w/o triton) or permeabilized with 0,1% triton prior to staining (+ triton). All bacteria positively stained with the α-*Salmonella* LPS antiserum were included for analysis and *S.* Tm GFP-fluorescence intensity (grey levels; y-axis) was determined by automated image analysis (**A, B**). Representative images of *S.* Tm^wt^ or *S.* Tm^avir^ in the cecal lumen expressing p*iroBCDE*-*gfp* (**C**) or p*sodB*-*gfp* (**D**) (green). The section was permeabilized with triton-x-100 and stained with α-*Salmonella* LPS antiserum (red) and phalloidin (blue). Scale bar: 10 µm.

We set out to analyze if *S.* Tm expresses *iroBCDE* in gut luminal neutrophils. To this end, we generated a GFP-reporter for the *iroBCDE* promoter (p*iroBCDE*-*gfp*). Expression of *iroBCDE* is regulated via Fur, induced upon iron starvation and repressed under iron excess (**Fig. S1**) [31]. Streptomycin-treated mice were infected with *S.* Tm^wt^ (n = 3) or *S.* Tm^avir^ (n = 3) containing p*iroBCDE*-*gfp* to analyze *gfp-*expression under control of *iroB* promoter under inflammatory and non-inflammatory conditions, respectively. Tissues were fixed and bacteria in different regions within the gut lumen were analyzed with respect to *gfp-*expression by automated image analysis. All bacteria positively stained with the α-*Salmonella* LPS antiserum (red) were analyzed with respect to GFP-fluorescence intensity (green). On average, 1’200 bacteria per mouse (≥ different cecum lumen tissue sections) were analyzed this way and we found a significantly lower GFP-intensity in *S.* Tm^wt^ as compared to *S.* Tm^avir^ infected mice (**Fig. 6A, left panel**). We hypothesized that our analysis did not include those bacteria residing within neutrophils as the tissue was not permeabilized prior to staining with the α-*Salmonella* LPS antiserum.

Therefore the same analysis was performed on sections that were permeabilized with 0.1% triton X 100 before α-*Salmonella* LPS staining. Now, the GFP-intensity of p*iroBCDE*-*gfp* expressing *S.* Tm was significantly higher in the lumen of inflamed mice (**Fig. 6A** **; compare left and right panels**). This suggested that p*iroBCDE*-*gfp* was particularly induced in the pathogen-subpopulation residing within the gut luminal neutrophils.

Expression of the *iroBCDE* promoter is under the control of the Fur-regulon and expressed under iron-limiting conditions [21]. Our results suggest that neutrophils in the inflamed gut lumen constitute an iron-limiting environment for bacteria. To verify this, we used a second GFP-reporter construct (p*sodC*-*gfp*). *SodC* encodes an iron-superoxide dismutase which is also regulated by Fur. However, *sodC* expression is induced in the presence of iron and is therefore regulated in the opposite fashion as *iroBCDE* [31](**Fig. S1**). Indeed we found that p*sodC*-*gfp* expression was significantly lower in the inflamed than in the non-inflamed gut (**Fig. 6B**). Triton permeabilization had no effect on the difference of p*sodC*-*gfp* expression between inflamed and non-inflamed gut.

This data extends earlier reports on iron limitation in the inflamed gut [22] by demonstrating that iron limitation pertains to extracellular bacteria as well as to *S.* Tm residing within gut luminal neutrophils. In conclusion these data show that *S.* Tm expresses *iroBCDE* in neutrophils. This may be due to general iron limiting milieu (i.e. due to increased lipocalin-2 expression by the mucosa and possibly also neutrophils) in the inflamed gut lumen. This indicates that the transmigrating neutrophils are still active in the gut lumen with respect to bacterial uptake and iron limitation in the phagocytic vacuole.

## Discussion

Non-typhoidal *Salmonella* spp. can exploit the host defense to compete against the intrinsic microbiota. Here, we characterized the interaction of NTS with neutrophils, an important cellular component of the innate host defense within infected tissues. In response to *S.* Tm infection, neutrophils are attracted to the site of infection. Within 10h after oral *S.* Tm^wt^ infection of streptomycin-treated mice, neutrophils can already be observed in the cecal mucosa at high numbers [18]. Furthermore, neutrophils even transmigrate into the gut lumen, frequently leading to crypt abscess formation.

It remained unclear what role neutrophils play for growth and survival of *S.* Tm in the gut lumen. Upon infection, *S.* Tm^wt^ grows at high titers (10^7^ to 10^9^ cfu/g) in the gut lumen despite the presence of luminal neutrophils. This condition holds up for at least 4 days without reducing *S.* Tm^wt^ numbers in the cecal lumen or in the feces [14]. This suggests that neutrophils are not able to eliminate the pathogen from the gut lumen. Similarly, a previous study showed that *S.* Tm can survive quite well within murine neutrophils [32]. Our data from the gentamicin-protection assay demonstrated that viable *S.* Tm can indeed be recovered from gut luminal neutrophils. Interestingly, the key virulence factor for *S.* Tm intracellular survival, TTSS-2, although expressed within neutrophils, seems to be dispensable for gut luminal survival of *S.* Tm. This was previously demonstrated in co-infection experiments competing *S.* Tm^wt^ with a mutant lacking functional TTSS-2 [14]. The *ttss-2* mutant colonized the inflamed gut equally well as the wild type up to day 4 p.i. As discussed below, the lack of a TTSS-2 mediated growth/survival benefit for the pathogen in the gut lumen might be explained by the short life-span of the gut luminal neutrophils.

Strikingly, up to ∼70% of the *S.* Tm bacteria were localized within neutrophils at day 1 p.i., as determined by fluorescence microscopy, suggesting that most bacteria in the gut lumen do actually encounter neutrophils (**Fig. 4B**). On the other hand, just 0.62% of the viable *S.* Tm in the cecal contents (as determined by plating) were actually protected against gentamicin. This apparent discrepancy could be explained if neutrophils had just a limited “active” lifetime upon transmigration into the intestinal lumen. This would be in line with the relatively short lifetime of murine neutrophils in bone marrow or after isolation from blood [33], [34]. However, the limited lifetime in the gut lumen should be long enough for the uptake (and possibly elimination) of bacteria localized right at the mucosal surface as indicated by the active phagocytosis of *S.* Tm^avir^ and the intracellular induction of the p*ssaG*-*gfp* and p*iroBCDE* reporter. Afterwards, the neutrophils would die off thus rendering the internalized bacteria prone to killing in the gentamicin protection assay. Nevertheless, many bacteria seem to remain associated with these dead/dying neutrophils (and can thus be detected within neutrophils by fluorescence microscopy). The long-lived nature of Gfp within the bacterial cytosol could explain why p*ssaG*-*gfp* and p*iroBCDE* -driven *gfp* expression can still be detected long after the active life of the neutrophil (incl. intracellular iron limitation) has ended by the host cell’s demise.

Most parts of the normal anaerobic microbiota are outcompeted by *S.* Tm^wt^ in the inflamed gut. Neutrophils have been proposed to modify the microbiota-pathogen interaction [35]. Neutrophils harbor an arsenal of antimicrobial defenses including oxidative burst and the generation of reactive oxygen species (ROS), antimicrobial peptides (calprotectin, lipocalin, lysozyme) and formation of neutrophils extracellular traps (NETs) [36], [37]. Most commensals would probably show a higher susceptibility to this variety of antimicrobial defenses. Deciphering the different possible contributions of neutrophils to the microbiota-pathogen competition in the gut lumen will be a challenging subject of future research.

Our data suggest that neutrophils may form a distinct compartment in the inflamed gut lumen. Thus, neutrophils could affect the lifestyle of pathogens as well as commensals by generating an intracellular compartment displaying specific physiological properties. We found that neutrophil-contained *S.* Tm up-regulated the promoter for *iroBCDE*. This suggests that gut luminal neutrophils limit iron availability to the engulfed bacteria. Furthermore, our data suggest that *S.* Tm responds to iron limitation experienced within the inflamed gut lumen by expressing salmochelin. Expression of *iroN*, the salmochelin-receptor has been shown to entail a competitive advantage for *S.* Tm in the inflamed gut as it overcomes inhibition by the antimicrobial protein lipocalin-2. Interestingly, lipocalin-2 is known to be expressed by human and murine neutrophils [30], [38]. This suggests that salmochelin expression may be important for *S.* Tm interaction and/or survival within gut luminal neutrophils.

The respiratory burst of neutrophils that transmigrate into the intestinal lumen during inflammation oxidizes endogenous sulfur compounds to generate a respiratory electron acceptor, tetrathionate [20]. It is tempting to speculate that this oxidation takes place in the neutrophil compartment. In this case, intracellular *S.* Tm may capitalize on the tetrathionate, available within neutrophils. In light of the data showing a defect of the *S.* Tm chemotaxis mutant to enter neutrophils, one could even speculate that the bacteria move along chemotactic gradients of such anaerobic electron acceptors released by luminal neutrophils. Future experiments will have to address this.

In conclusion, we identified gut luminal neutrophils as novel niche for *S*. Tm in the infected gut. Our results suggest that this intra-neutrophil niche may affect *S.* Tm lifestyle. Future studies will have to address the exact underlying mechanisms as well as the role of neutrophils in microbiota-pathogen competition within the inflamed gut.

## Materials and Methods

### Construction of Plasmids

Promoter regions of *iroBCDE* and *sodB* were amplified with the primer pair iroBCDE_F-XbaI/iroBCDE_R-BamHI and sodB_F-XbaI/sodB_R-BamHI, respectively as described before [31] and cloned into pM968 ([18]; promoterless pBAD24-derivative) via *Xba*I and *Bam*HI to generate p*iroBCDE*-*gfp* and p*sodB*-*gfp,* respectively.

### Animal Experiments

All mice used in the study were on C57Bl/6 background and bred at the Rodent Center at the ETH Zürich and the Max-von Pettenkofer Institute under SPF conditions in individually ventilated cages. For *S.* Tm infections, mice were treated with streptomycin (20 mg/animal 20h prior to *Salmonella* infection) and infected by oral gavage with 5×10^7^ cfu *S.* Tm or mixtures of *S.* Tm as described [5]. Live bacterial loads in the cecal content were determined by plating on MacConkey-agar (Oxoid) with respective antibiotics (streptomycin 100µg/ml; kanamycin 30 µg/ml; chloramphenicol 30 µg/ml; ampicillin 100 µg/ml; tetracycline 12 µg/ml). Histology was done at necropsy.

For *ex vivo Salmonella* infection of cecal tissue, strain *S.* Tm^avir^ with the YFP plasmid was cultivated under aeration and appropriate antibiotic therapy and constant agitation in high-salt LB medium (300 mM NaCl) until an OD_600_ of 0.8–1.0 was reached. Bacteria were harvested by centrifugation (5000 r.p.m., 5 min) and once washed in PBS. About 1×10^7^ cfu were administered in a volume of 50 µl. The nuclei were stained with DAPI at concentrations of 0.2 µg ml^−1^ and with lipophilic styryl dye FM 4-64 (Invitrogen; 10 µg ml^−1^). The tissue was mounted on an organ holder and analysed using an inverse Leica SP5 confocal laser scanning microscope (Leica Microsystems, Mannheim, Germany) in a 37°C chamber.

### Ethics Statement

All animal experiments were approved (license 201/2004 and 201/2007 Kantonales Veterinäramt Zürich) and the Regierung von Oberbayern and performed according to local guidelines (TschV, Zurich), the Swiss animal protection law (TschG) and the Deutsches Tierschutzgesetz.

### Immunofluorescence

Cecal tissues were recovered and treated as described [19]. Briefly, the tissue was fixed in paraformaldehyde (4% in PBS, pH 7.4 overnight, 4°C), washed with PBS, equilibrated in PBS (20% sucrose, 0.1% NaN_3_ overnight, 4°C), embedded in O.C.T. (Sakura, Torrance, CA), snap-frozen in liquid nitrogen and stored at −80°C. Cryosections (7 µm) were air-dried for 2 h at room temperature, fixed in 4% paraformaldehyde (5 min). Tissue was optionally permeabilized for 30 minutes in PBS 0.1% triton X 100. Sections were washed and blocked in 10% (w/v) normal goat serum in PBS for 1h. *S. Tm* were detected by staining for 1h with a polyclonal rabbit α-*Salmonella*-O-antigen group B serum (factors 4, 5; Difco; 1∶300) and Cy3-conjugated secondary goat-α-rabbit antibody (Jackson). Neutrophils were stained with rat- α-CD18 antibody (BD-Pharmingen) and Cy3-conjugated secondary goat-α-rat antibody (Jackson). DNA was stained with Sytox-green (1∶10,000; Invitrogen). Sections were mounted with Vectashield (Vector laboratories). Imaging was done using a Leica SP5 confocal laser scanning microscope (Leica Microsystems, Mannheim, Germany; Fig. 1,3) or an Axiovert 200M inverted microscope (Zeiss; Fig. 4,5 and 6).

For immunofluorescent staining of extracellular *S.* Tm in cecum sections (Figure 4, 5 and 6), non-permeabilized cecum sections (7µm) were stained with rabbit α-*Salmonella*-O-antigen group B serum (factors 4, 5; Difco; 1∶300) and Cy3-conjugated secondary goat-α-rabbit antibody (Jackson). The fraction of intracellular GFP^+^ *S.* Tm was determined as (GFP^+^/ GFP^+^ LPS^+^) × 100%. 200–800 bacteria per group were included into the analysis.

For automated image analysis (Fig. 6), confocal stacks of immunofluorescence stained cecal tissue were recorded with an Ultraview confocal head (PerkinElmer), a krypton argon laser (643-RYB-A01; Melles) and Plan Neofluar 64x oil objective, aperture setting 1.25 (Zeiss). Confocal stacks were recorded with Volocity software (Improvision Ltd.) with a distance of 0.5µm between the layers. From each cecum lumen, a total of 6 pictures were taken. GFP intensity was measured automatically with the Volocity Quantitation tool using following conditions: (1) selection of all red bacteria (2) mean green intensity of all selected bacteria from 300 to 1000 was considered for analysis.

### Gentamicin Protection Assay

Cecum content of mice was harvested at day 1 post infection with *S.* Tm^wt^. The content was resuspended in PBS and split into 4 fractions. The fractions were either treated with triton-X-100 (0.1%) to lyse all intact host cells or with gentamicin (400 µg/ml) to kill all extracellular bacteria or with both (triton-X-100 and gentamicin) to kill all bacteria, or left untreated, as control. Samples were incubated at 37°C, shaking for 30 minutes. Samples were centrifuged (10,000 rpm; 2 min) and washed twice with PBS. Samples were then treated with 1% triton-X-100 and plated on LB containing 100 µg/ml streptomycin in appropriate dilutions.

### Statistical Analysis

Statistical analysis was performed using the exact Mann-Whitney U Test (Graphpad Prism Version 5.01). P-values less than 0.05 (2-tailed) were considered statistically significant.

## Supporting Information

Figure S1 **Iron responsiveness of the two reporter constructs p** ***iroBCDE*** **-** ***gfp*** **and p** ***sodB*** **-** ***gfp.*** The histidin-prototrophic *S.* Tm^avir^ strain M960 harboring either p*iroBCDE*-*gfp* (**left**) or p*sodB*-*gfp* (**right**).The bacteria were grown in M9 medium with glucose (2 g/l) over night and diluted 1∶20 in fresh M9 media containing either the iron chelator diethylene triamine pentaacetic acid (**A**, DTPA; 0.1 µM, 1 µM, 10 µM), no supplement, or Fe(III) chloride (**B**, FeCl_3_; 1 µM, 10 µM, 100 µM) to vary iron availability. The subcultures were incubated for 4h at 37°C on the rotating wheel and then submitted to FACS analysis on an LSR II instrument (BD Biosciences). Data analysis was performed with FlowJo 7.5 (Tree Star, Inc.). The bacterial population was gated in FCS/SSC and GFP fluorescence was measured in FL1 channel (histogram).
(PDF)

Movie S1 **Time-lapse microscopy of neutrophils in the lumen of an explanted cecum.** Streptomycin-treated mice LysM^gfp^ +/− mice were infected with *S.* Tm^wt^ and sacrificed 24 h post infection as described in main Figure 3. The cecum was explanted and infected *ex vivo* with *yfp* expressing *S.* Tm^avir^, stained with DAPI (DNA; blue) and FM 4–64 (epithelial membranes; red), mounted on an organ holder and analyzed by confocal real time microscopy of *S.* Tm and GFP^+^ neutrophils. The videos show motile neutrophils (GFP; green) in the lumen of the explanted cecum. Scale bars: 10 µm.
(AVI)

Movie S2 **Time-lapse microscopy of neutrophils in the lumen of an explanted cecum.** Streptomycin-treated mice LysM^gfp^ +/− mice were infected with *S.* Tm^wt^ and sacrificed 24 h post infection as described in main Figure 3. The cecum was explanted and infected *ex vivo* with *yfp* expressing *S.* Tm^avir^, stained with DAPI (DNA; blue) and FM 4–64 (epithelial membranes; red), mounted on an organ holder and analyzed by confocal real time microscopy of *S.* Tm and GFP^+^ neutrophils. The videos show motile neutrophils (GFP; green) in the lumen of the explanted cecum. Scale bars: 10 µm.
(AVI)
