# Supplementary figures and images for: Salmonella Transiently Reside in Luminal Neutrophils in the Inflamed Gut
Source: PLoS One. 2012 Apr 6;7(4):e34812. doi: 10.1371/journal.pone.0034812 (PMC3321032; doi:10.1371/journal.pone.0034812)

# Loetscher et al., Fig. S1. Iron-dependent regulation of *piroBCDEN* and *psodB*

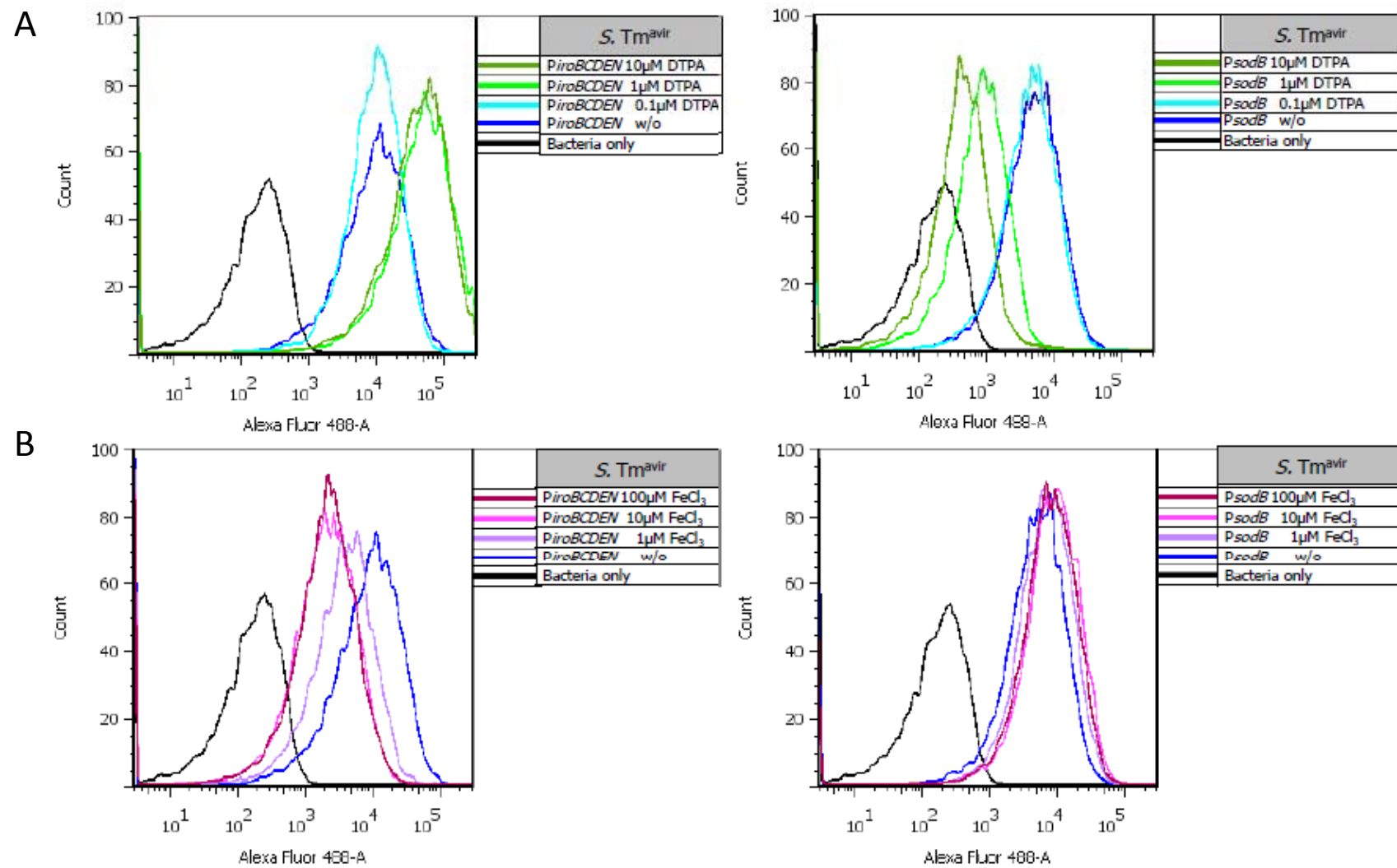

Supplement: Figure S1 — Iron responsiveness of the two reporter constructs p iroBCDE - gfp and p sodB - gfp. The histidin-prototrophic S. Tmavir strain M960 harboring either piroBCDE-gfp (left) or psodB-gfp (right).The bacteria were grown in M9 medium with glucose (2 g/l) over night and diluted 1∶20 in fresh M9 media containing either the iron chelator diethylene triamine pentaacetic acid (A, DTPA; 0.1 µM, 1 µM, 10 µM), no supplement, or Fe(III) chloride (B, FeCl3; 1 µM, 10 µM, 100 µM) to vary iron availability. The subcultures were incubated for 4h at 37°C on the rotating wheel and then submitted to FACS analysis on an LSR II instrument (BD Biosciences). Data analysis was performed with FlowJo 7.5 (Tree Star, Inc.). The bacterial population was gated in FCS/SSC and GFP fluorescence was measured in FL1 channel (histogram). (PDF) [file pone.0034812.s001.pdf]
